# Flexible Cyclic Immunofluorescence (cyCIF) Using Oligonucleotide Barcoded Antibodies

**DOI:** 10.3390/cancers15030827

**Published:** 2023-01-29

**Authors:** Nathan P. McMahon, Jocelyn A. Jones, Ashley N. Anderson, Matthew S. Dietz, Melissa H. Wong, Summer L. Gibbs

**Affiliations:** 1 Biomedical Engineering Department, Oregon Health & Science University, Portland, OR 97201, USA; 2 Department of Cell, Development & Cancer Biology Department, Oregon Health & Science University, Portland, OR 97201, USA; 3 Knight Cancer Institute, Oregon Health & Science University, Portland, OR 97201, USA

**Keywords:** cyclic immunofluorescence (cyCIF), spatial proteomics, DNA barcoded antibodies, photocleavable linkers

## Abstract

**Simple Summary:**

Advances in our understanding of the complex spatial interactions between tumor epithelia and tumor microenvironmental cells have been driven by highly multiplexed imaging technologies. These techniques are capable of labeling many more biomarkers than conventional immunostaining methods. However, multiplexed imaging techniques suffer from low detection sensitivity, cell loss—particularly in fragile samples—and challenges with antibody labeling. Herein, we developed and optimized a DNA antibody barcoding strategy for cyclic immunofluorescence (cyCIF) that can be amplified to increase the detection efficiency of low-abundance antigens. Stained fluorescence signals can be readily removed using ultraviolet light treatment, preserving tissue and fragile cell sample integrity.

**Abstract:**

Advances in our understanding of the complex, multifaceted interactions between tumor epithelia, immune infiltrate, and tumor microenvironmental cells have been driven by highly multiplexed imaging technologies. These techniques are capable of labeling many more biomarkers than conventional immunostaining methods. However, multiplexed imaging techniques suffer from low detection sensitivity, cell loss—particularly in fragile samples—, and challenges with antibody labeling. Herein, we developed and optimized an oligonucleotide antibody barcoding strategy for cyclic immunofluorescence (cyCIF) that can be amplified to increase the detection efficiency of low-abundance antigens. Stained fluorescence signals can be readily removed using ultraviolet light treatment, preserving tissue and fragile cell sample integrity. We also extended the oligonucleotide barcoding strategy to secondary antibodies to enable the inclusion of difficult-to-label primary antibodies in a cyCIF panel. Using both the amplification oligonucleotides to label DNA barcoded antibodies and in situ hybridization of multiple fluorescently labeled oligonucleotides resulted in signal amplification and increased signal-to-background ratios. This procedure was optimized through the examination of staining parameters including staining oligonucleotide concentration, staining temperature, and oligonucleotide sequence design, resulting in a robust amplification technique. As a proof-of-concept, we demonstrate the flexibility of our cyCIF strategy by simultaneously imaging with the original oligonucleotide conjugated antibody (Ab-oligo) cyCIF strategy, the novel Ab-oligo cyCIF amplification strategy, as well as direct and indirect immunofluorescence to generate highly multiplexed images.

## 1. Introduction

Highly multiplexed imaging techniques have risen in popularity as the biological significance of spatial proteomics has been appreciated. This is particularly evident in the study of cancer, where these imaging tools are capable of measuring the expression and spatial distribution of proteins that define unique populations of tumor epithelia, immune infiltrate, and tumor microenvironmental (TME) cells [1]. Substantial advances in therapeutic strategy design have been realized in immunotherapy in part through the utilization of multiplexed technologies to assist in target identification and subsequent therapeutic response to inform on therapeutic efficacy and resistance, as well as the role of the TME in both of these biological phenomena [2,3]. Additionally, recent multiplexed proteomic profiling of a patient cohort of advanced-stage colorectal cancer (CRC) identified nine distinct cellular neighborhoods within the immune TME. Notably, this analysis revealed that the presence of a cellular neighborhood enriched with granulocytes, with only programmed death ligand (PD-1)+ and CD4+ T cells being positively correlated with survival amongst high-risk patients [4]. Intraductal papillary mucinous neoplasms (IPMNs), a precursor to pancreatic ductal adenocarcinoma (PDAC), have revealed that the spatial proximity between epithelial cells and cytotoxic T cells is predictive in determining which IPMN patients will develop PDAC, aiding in understanding disease progression and potentially improving therapeutic selection for these patients [5]. Further development of multiplexed imaging techniques to alleviate the difficulties associated with low detection sensitivity, cell loss—particularly in fragile samples, and challenges with antibody labeling—will facilitate comprehensive spatial proteomics in a variety of tissue types and disease states to understand disease progression and improve therapeutic outcomes for patients.

There are currently two main modalities for highly multiplexed imaging that use either (1) antibody staining (i.e., immunohistochemistry [IHC] or immunofluorescence [IF]) or (2) mass spectrometry imaging (MSI) with rare earth metal-labeled antibodies [6,7,8,9,10,11,12,13,14,15,16,17]. Cyclic antibody-based approaches are broadly performed by repeated staining, imaging, and signal removal through fluorophore bleaching [9,14,15] or antibody stripping [17,18,19]. These workflows can be integrated into histopathological workflows using existing microscopy tools and, therefore, have seen broad adoption. However, repeated, lengthy antibody incubation steps limit the throughput of these techniques. Additionally, detection sensitivity is limited due to the semi-quantitative nature of IHC and decreased fluorescence signal produced by the fluorophore-labeled antibodies necessary for cyclic IF in comparison to conventional indirect IF. In contrast, MSI (e.g., MIBI [6,13], CyTOF [10], etc.), is performed by applying all antibodies in a single step as a “master-mix” and imaging for all markers is performed in a single scan based on the unique molecular weight labels. However, by comparison to cyclic IF or IHC that can be detected using microscopy, instrumentation for MSI is far less available, particularly in histopathology labs, and thus its use is not as widespread or readily translated for clinical use. Additionally, spatial resolution is limited by the scanning laser spot size, hampering the detection of intracellular structures, and in some instances individual cells. Overcoming the limitations of these techniques, oligonucleotide “barcoded” antibody-based highly multiplexed fluorescence imaging methodologies have emerged as a hybrid solution. In these techniques, all DNA-barcoded antibodies stain the sample in a single step as a “master-mix” similar to MSI, while single-cell resolution and quantitative fluorescence imaging are achieved as in conventional antibody-based highly multiplexed IF approaches. Additionally, antibody barcoding techniques (e.g., DNA exchange imaging [20], NanoString [21,22], and CODEX [23]) have demonstrated the ability for highly multiplexed immunostaining using non-destructive signal removal techniques.

While all of these techniques have demonstrated capability for high dimensional image generation, they lack an amplification system to increase signal for markers with low abundance. In response a number of methods for fluorescence signal amplification have been developed (e.g., a hapten-based modified multiplex [24] and tyramide signal amplification [25]), where detection sensitivity has been demonstrated to be up to 10× greater than that of conventional indirect IF. These techniques meet the analytical need for increased detection sensitivity but are limited to a maximum of seven biomarkers, which is substantially less than current cyclic immunostaining platforms. Alternatively, fluorescence-based methods of in situ hybridization to measure mRNA expression in tissue have been developed that utilize the mechanism of hybridization chain reaction for signal amplification [26,27]. In these methods, oligonucleotide probes hybridize to a complementary mRNA molecule followed by incubation of fluorophore-labeled oligonucleotide hairpins that self-assemble into a tethered amplification system. Importantly, this system has been used to measure up to 24 distinct mRNA sequences from a single tissue sample representing improved multiplexing over antibody-based signal amplification methods [28]. However, this oligonucleotide-based hybridization system has not yet been successfully translated to antibody-based immunofluorescence imaging, but offers a potential avenue for robust signal amplification and high dimensional imaging.

**Figure 1:**
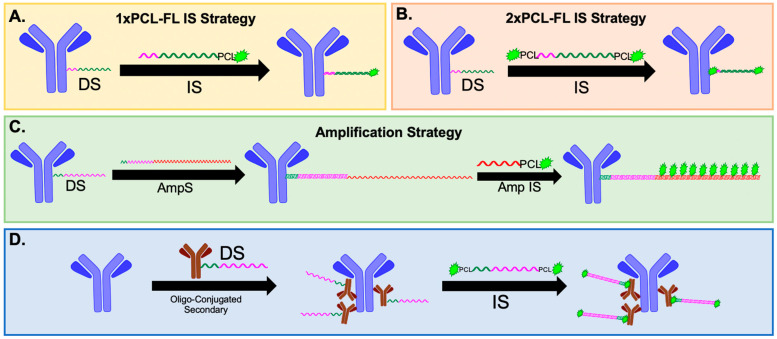
**Ab-oligo cyCIF strategies.** Multiple methods of fluorescent labeling of Ab-oligo conjugates have been investigated including (**A**) a complementary imaging strand (IS, 26 nt) with a single fluorophore and photocleavable linker (PCL) and (**B**) a complementary IS (26 nt) with two fluorophores and two PCLs that hybridize to the docking strand (DS, 28 nt) on the antibody. (**C**) An amplification strategy has been developed and optimized that utilizes an amplification strand (AmpS, 198 nt) that hybridizes with DS (28 nt) and has an unhybridized region that can hybridize up to ten amplification IS (Amp IS, 15 nt) that each contain a fluorophore and PCL for increased signal intensity. (**D**) The oligonucleotide conjugation strategy was also extended to secondary antibodies to permit Ab-oligo cyCIF of sensitive primary antibodies where DS (28 nt) are conjugated to secondary antibodies and detected using complementary 2× PCL-FL IS (26 nt).

We have previously reported on our optimized oligonucleotide conjugated antibody (Ab-oligo) cyclic immunofluorescence (cyCIF) platform which utilizes docking strand (DS) labeled antibodies (Ab-oligos) and complementary fluorophore-labeled oligonucleotides, termed an imaging strand (IS), which hybridize for in situ antigen labeling. The IS is fluorophore-labeled through a photocleavable linker (PCL) facilitating signal removal through exposure to UV light [29]. Herein, we demonstrate that the signal-to-background ratio (SBR) for Ab-oligo cyCIF is improved by going from a single fluorophore on each IS (1× PCL-FL IS, Figure 1A) to two fluorophores on each IS (2× PCL-FL IS, Figure 1B). We also report on the development of an Ab-oligo cyCIF imaging strategy to amplify the Ab-oligo fluorescence signal intensity (Figure 1C). In this Ab-oligo amplification strategy, a single-stranded oligonucleotide sequence, termed the amplification strand (AmpS), consists of 198 nucleotides (nt), 28 of which completely hybridize with the complementary 28 nt Ab-oligo DS. Then, fluorescence labeling is performed by staining with amplification IS (Amp IS), which is complementary to the non-hybridized region of the AmpS strand. The length of the AmpS strand is sufficient for up to ten fluorophores to label a single DS, amplifying the maximum signal intensity compared to our previous fully complementary IS. Additionally, we have also extended our oligonucleotide barcoding strategy to secondary antibodies which can be used for indirect IF staining (Figure 1D) at the beginning of the cyCIF studies, permitting the use of antibodies that lose binding affinity upon oligonucleotide conjugations. Combining these oligonucleotide labeling strategies (Figure 1B–D) with standard IF permits a flexible cyCIF strategy that can quantify the spatial proteomics of a variety of antigen targets.

As described herein, we validate our flexible cyCIF methodology, demonstrating its capability to generate highly multiplexed images. We detail the development and validation of the Ab-oligo amplification strategy, showing fluorescence signal equivalent or greater than conventional indirect IF. We also demonstrate the flexibility of the Ab-oligo cyCIF platform where multiple strategies can be deployed simultaneously on the same sample to selectively amplify low-abundance antigens, such as phosphoproteins. Additionally, we show that the Ab-oligo cyCIF platform is superior to conventional fluorophore-labeled antibodies that must be removed by harsh methods for delicate samples, such as blood smears that lack the structural integrity of an extracellular matrix. Herein, we show that blood smear samples can be imaged for many cycles using Ab-oligo cyCIF with minimal sample degradation compared to more conventional bleaching cyCIF. Our optimized Ab-oligo cyCIF amplification strategy overcomes several challenges associated with other highly multiplexed platforms with the overall goal of providing sufficient sensitivity to continue to unravel the multifaceted interactions that drive the hallmarks of cancer.

## 2. Materials and Methods

### 2.1. Study Design

The goals of this study were to optimize methods to increase cyclic immunofluorescence (cyCIF) staining signal, method flexibility, and ability to stain fragile samples, such as blood smears. To this end, we demonstrate proof-of-concept flexible cyclic methods that expand on our previously published cyCIF platform, including a method for signal amplification to visualize low-abundance antigens, such as phosphorylated proteins, and the development of oligonucleotide-conjugated secondary antibodies to expand the platform to antibodies where direct oligonucleotide conjugation was not feasible (Figure 1). Verification of antibody specificity was determined based on prior reports using the same clones and predicted cell localization [29,30,31]. Where appropriate, all experiments were performed with the following samples and controls: (1) gold standard indirect immunofluorescence (IF: unconjugated primary + fluorophore-labeled secondary), (2) a fluorophore-labeled secondary antibody without primary antibody staining as a negative control, (3) an Ab-oligo conjugate + 2× photocleavable linker (PCL) labeled with a fluorophore (FL) imaging strand (IS), (4) 2× PCL-FL IS only without Ab-oligo conjugate as a negative control, (5) an Ab-oligo conjugate + amplification strand (AmpS) + amplification imaging strand (Amp IS), (6) AmpS + Amp IS without Ab-oligo conjugate as a negative control, (7) an unconjugated primary antibody + oligonucleotide-conjugated secondary + 2× PCL-FL IS, (8) and an oligonucleotide-conjugated secondary + 2× PCL-FL IS without primary antibody as a negative control. All antibody clones and conjugated antibodies were verified for specificity using these staining strategies prior to use in any additional experiments. Primary antibodies and Ab-oligos were all used at a final concentration of 15 μg/mL, whereas all secondary antibodies, 2× PCL-FL IS, AmpS, and Amp IS, were used at a 350 nM concentration, or as specified below. All cell and tissue samples were first fixed, as described below, at the time they reached confluence or at collection. Additionally, unconjugated primary antibodies and Ab-oligos were post-fixed in place prior to the addition of secondary antibodies or IS. The secondary fixation step was used to ensure antibody preservation across multiple rounds of staining and to maintain consistent staining methods between experiments. Additionally, to maintain consistency between staining experiments, all staining reagents, including IS, Amp IS, and secondary antibodies, were diluted in the same dilution buffers as those found to be optimal for the IS.

### 2.2. Human Subjects

Human peripheral blood samples from patients with colorectal cancer (CRC) and formalin-fixed paraffin-embedded (FFPE) CRC and breast tissue sections were collected and analyzed under approved protocols in accordance with the ethical requirements and regulations of the Oregon Health and Science University (OHSU) institutional review board (IRB #5169). Samples were obtained from the OHSU BioLibrary under our approved IRB.

### 2.3. Animal Care and Use

All animal experiments were approved by the OHSU Institutional Animal Care and Use Committee (IACUC). All mice were housed in an AAALAC-certified OHSU vivarium and supplied with food, water, and daily inspection to monitor for pain or distress for the duration of experimentation. Mice were placed on a chlorophyll-free diet (Animal Specialties, Inc., Hubbard, OR, USA) one week prior to tumor resection. All rodent surgical procedures described herein were performed under full anesthesia composed of a 90/10 mixture of ketamine/xylazine. Ketamine (Hospira Inc., Lake Forest, IL, USA) was administered at a dose of 100 mg/kg and xylazine (AnaSed, Shenandoah, IA, USA) was administered at a dose of 10 mg/kg by intraperitoneal (IP) injection. The toe pinch method was employed to verify the depth of anesthesia prior to the commencement of any surgical procedures. The standard method of euthanasia for mice was the inhalation of carbon dioxide under full anesthesia at the end of each experiment. Euthanasia was confirmed by physical examination to ensure the cessation of heartbeat and respiration and is consistent with the recommendations of the Panel on Euthanasia of the American Veterinary Medical Association.

## 3. Antibody and Sample Preparation

### 3.1. Fluorophore-Labeled Primary and Secondary Antibodies

Mitogen-activated protein kinase (MEK) Alexa Fluor 488 (MEK-AF488, Abcam, Cambridge, MA, USA) and mouse reactive CD45 labeled with Alexa Fluor (CD45-AF647, Cell Signaling Technology, Danvers, MA, USA) primary antibodies (Table 1) were purchased pre-labeled with the described Alexa Fluor (AF) dyes and used for the final round of cyCIF staining. CD45 was previously validated to be specific to only mouse antigens to ensure no cross-reactivity with human tumor cells in cell line-derived xenograft (CDX) model systems [32]. Unconjugated primary antibodies and secondary antibodies, including donkey anti-rabbit and donkey anti-mouse (Jackson ImmunoResearch Inc., West Grove, PA, USA), were used for indirect IF and corresponding secondary-only negative control staining studies throughout the reported studies. As needed, secondary antibodies were conjugated to AF fluorophores (ThermoFisher Scientific, Waltham, MA, USA) via a standard *N*-hydroxysuccinimide (NHS) ester chemistry, as previously described [33,34,35].

### 3.2. The Generation of an Oligonucleotide Conjugated Primary and Secondary Antibodies (Ab-Oligo)

Oligonucleotide-conjugated primary antibodies were generated, as previously reported [29]. In brief, primary antibodies to human E-Cadherin (E-Cad), cytokeratin 5 (CK5), CK7, CK8, CK14, CK19, epidermal growth factor receptor (EGFR), phosphorylated-EGFR (pEGFR), AKT, phosphorylated-AKT (pAKT), phosphorylated-MEK (pMEK), phosphoinositide-3-kinase (PI3K), cleaved caspase-3 (CC3), Ki67, human epidermal growth factor receptor 2 (HER2), alpha-smooth muscle actin (αSMA), CD4, cytochrome c oxidase subunit IV (CoxIV), CD68, programmed cell death protein 1 (PD1), proliferating cell nuclear antigen (PCNA), androgen receptor (AR), vimentin (VIM), estrogen receptor (ER), and progesterone receptor (PR) were purchased from AbCam (Cambridge, UK), as well as Cell Signaling Technology (Danvers, MA, USA), Biolegend (San Diego, CA), Bio-Rad (Hercules, CA, USA), and ThermoFisher Scientific (Waltham, MA, USA) detailed in Table 1. Donkey anti-mouse secondary antibody from Jackson ImmunoResearch (West Grove, PA, USA) was used for the generation of the oligonucleotide-conjugated secondary antibody. A unique dibenzocyclooctyne-terminated (DBCO) single-stranded oligonucleotide (docking strand [DS], 28 nucleotides [nt] in length), was used to label each antibody (Integrated DNA Technologies [IDT], Coralville, IA, USA). Antibody modification and oligonucleotide conjugation were completed with the SiteClick^TM^ Antibody Azido modification kit (ThermoFisher Scientific), as previously described [29,31].

### 3.3. Optimal Docking Strand (DS) Oligonucleotide Sequence Design for Antibody Conjugation

Docking strands (DS) were evaluated for the potential to form secondary structures using the IDT OligoAnalyzer^TM^ Tool. Briefly, AmpS oligonucleotide sequences were analyzed with the hairpin function where the potential for secondary structure formation was calculated. If an oligonucleotide had a high potential for secondary structure formation, nucleotides from the portion of the AmpS which were complementary to the DS and involved in the hairpin were iteratively changed and tested until no hairpin structure was predicted. The resultant no hairpin AmpS was used to generate the new complementary sequence, or optimized DS, using the online Reverse Complement tool (https://www.bioinformatics.org/sms/rev_comp.html, accessed on 1 April 2019). Three AmpS sequences, including KpnIB, SalIC, and PvuIIA, were identified to have strong potential for secondary structure formation and were re-designed using the explained method to reduce the probability of secondary structure formation. The new, optimized DS sequences were conjugated to the trastuzumab (Herceptin^®^, Genentech, San Francisco, CA, USA) primary antibody, as described above. Fixed SK-BR-3 cells were stained with trastuzumab using conventional indirect IF with the unconjugated trastuzumab antibody, the trastuzumab Ab-oligo conjugate with the original DS design, or the trastuzumab Ab-oligo conjugate with the new DS design to quantitatively assess the utility of the oligonucleotide redesign strategy.

### 3.4. Mice Xenografts

The human epidermoid carcinoma cell line A431, the EGFR-mutated non-small cell lung cancer (NSCLC) cell line HCC827, and the estrogen receptor-positive human breast cancer cell line MCF7 transfected with human epidermal growth factor 2 (HER2/NEU, MCF7-HER2), [33,35,36,37,38,39] were cultured in DMEM 1× (Gibco/Thermo Fisher Scientific) with 10% fetal bovine serum (FBS, Seradigm, Sanborn, NY, USA) and 1% Penicillin-Streptomycin-Glutamine (Thermo Fisher Scientific). HCC827 cells were purchased from ATCC (Manassas, VA, USA). The A431 and MCF7-HER2 cells were a generous gift from Dr. Kimberley Samkoe (Dartmouth College, Hanover, NH, USA). All cell lines were grown to ~ 90% confluence prior to use for xenograft implantation.

Athymic nude mice (Homozygous 490, Charles River Laboratories, Wilmington, MA, USA) were purchased at 32–38 days old. After at least 48 hours (h) of acclimation, a total of two mice per cell line were subcutaneously implanted with A431, HCC827, or MCF7-HER2 xenografts, described briefly as follows. Cells were trypsinized by covering in 0.25% 1× Trypsin-ethylenediaminetetraacetic acid (Trypsin-EDTA, ThermoFisher Scientific), counted, and resuspended in their appropriate growth media to a concentration of 2 × 10^7^ cells/mL. The mice were then implanted subcutaneously with cells into each rear flank at a final concentration of 1 × 10^6^ cells/flank in 50% v/v Matrigel (Corning Inc., Corning, NY, USA), resulting in two tumors/mouse. The mice were monitored daily after implantation for tumor growth. The tumors were allowed to grow to a maximum size of 1 cm^3^, with growth times varying for each cell line (A431: ~2 weeks; MCF7-HER2: ~4–6 weeks, HCC827: ~7–8 weeks). All xenografts were resected when tumors reached a maximum size of 1 cm^3^ and flash frozen in an optimal cutting temperature (OCT) compound (Fisher Scientific). MCF7-HER2 xenografts were sent to OHSU’s Histology Shared Resource (HSR), where blocks were thawed and re-embedded in paraffin.

A431 and HCC827 were selected to serve as tissues positive for EGFR signaling pathway proteins and, due to rapid tissue preservation via flash freezing after tissue harvest, phosphorylated proteins were well-preserved. Tissues were flash-frozen immediately following harvest to minimize phosphorylated protein degradation. MCF7-HER2 cells were selected due to the known positive expression of HER2 and, for testing Ab-oligo cyCIF methods and reagents on FFPE tissue, the conventional fixation method in a clinical research setting was used.

### 3.5. Tissue and Cell Samples

The HER2-positive breast cancer cell line, SK-BR-3, was cultured in McCoy’s 5A (Gibco/Thermo Fisher Scientific) with 10% FBS and 1% Penicillin-Streptomycin-Glutamine. SK-BR-3 cells were seeded onto 96-well glass bottom plates (Cellvis, Mountain View, CA, USA) at 10,000 cells per well and grown to ~70% confluence for ~60 h at 37 °C and 5% CO_2_. Cells were fixed for 15 minutes (min) in 4% paraformaldehyde (PFA, Sigma Aldrich, St. Louis, MO, USA). PFA was removed and cells were washed with 1× PBS, pH 7.4 (3 × 5 min) and stored with 0.05% sodium azide (NaN_3_) at 4 °C until needed for staining. Prior to staining, the cells were washed with PBS (3 × 5 min) to remove NaN_3_. The cells were then permeabilized in a 0.5% polyoxyethylene(10) octylphenyl ether (Triton X-100, Millipore, Burlington, MA, USA) for 15 min followed by washing in PBS (3 × 5 min).

Peripheral blood mononuclear cell (PBMC) slides from patients with CRC were processed by collecting 10 mL of peripheral blood in heparinized vacutainer tubes (BD Biosciences, Franklin Lakes, NJ, USA) and diluted 1:2 with PBS pH 7.4 solution. PBMCs were isolated using density centrifugation with Ficoll-Paque PLUS (GE Healthcare, Chicago, IL, USA), as previously published [40]. Ficoll (12 mL) was layered on top of diluted blood and then centrifuged for 20 min at 800× *g* with no brake. Isolated PBMCs were resuspended in buffer and then adhered to poly-D-lysine (1 mg/mL, Millipore, Burington, MA, USA) coated slides (ThermoFisher Scientific) at 37 °C for 15 min. Cells were fixed with 4% PFA for 5 min, permeabilized with 0.5% Triton X-100 for 10 min, and fixed again with 4% PFA for 10 min. PBMC slides were then dehydrated in a series of ethanol baths (3 min each in 70%, 95%, and 100% ethanol) and stored at 4 °C until used for staining.

Fresh frozen mouse xenograft tissues were sectioned at 10 μm and captured onto 1 × 3-inch Superfrost Plus slides (ThermoFisher Scientific). Frozen tissue sections were incubated in 2% PFA for 15 min and then washed with 1× PBS, pH 7.4 (3 × 5 min). The tissue was then permeabilized using 1× PBS, pH 7.4 + 0.3% Triton X-100 for 15 min and washed with pH 7.4 PBS (3 × 5 min).

Archival clinical FFPE CRC and normal breast sections were provided by the OHSU Knight Biolibrary. After the resection of the tumor or normal tissue, fixation methods were performed per standard pathology operations, as previously described [30]. FFPE blocks of MCF7-HER2 xenografts, as well as cell buttons generated from MDA-MB-468 breast cancer cell lines, were generously provided by Dr. Koei Chin (OHSU, Portland, OR, USA). All FFPE tissue blocks were sectioned at 5 μm and captured onto 1 × 3-inch Superfrost Plus slides, which were baked at 65 °C in a hybridization oven for 30 min overnight prior to staining.

## 4. Staining Methods

### 4.1. Antigen Retrieval for FFPE Samples

Antigen retrieval was performed as previously described [29,31]. Briefly, FFPE sections were deparaffinized in xylenes followed by gradual rehydration in ethanol and water solutions. The deparaffinized tissue was rinsed in 100% deionized water (diH_2_O), followed by a wash in 1× PBS, pH 7.4 for 10 min. A two-step antigen retrieval procedure was performed in a Pascal Pressure Cooker (Dako, Santa Clara, CA, USA). Solutions of 10 mM sodium citrate, pH 6.0, 1× tris hydrochloride (HCl), pH 8.0, and diH_2_O were placed in the pressure cooker in three individual plastic buckets immersed in 500 mL of diH_2_O, which covered the bottom of the chamber. The slides were placed in the sodium citrate buffer and the pressure cooker lid was secured. The temperature was increased to 125 °C over 15 min, where the pressure reached ~15 psi. The temperature and pressure were then allowed to decrease to 90 °C and 0 psi, respectively, over ~25 min. The residual pressure was released as the lid was removed and the slides were rinsed in the hot diH_2_O. The slides were immediately placed in the hot tris-HCl buffer, where they were incubated for 10 min. The slides were then transferred back to the hot diH_2_O and brought back to room temperature by the slow addition of water to the vessel. After 5 min, the slides were washed in PBS and pH 7.4 at RT for an additional 5 min.

### 4.2. Conventional Antibody and Ab-Oligo Staining

The primary antibody or Ab-oligo conjugate incubation was performed, as previously reported for permeabilized cells [40], permeabilized frozen tissue [41], or FFPE tissue [29], and is described briefly as follows. Cells or tissue sections were blocked for 30 min in Ab-oligo blocking and dilution buffer which contained 2% bovine serum albumin (BSA, bioWORLD, Dublin, OH, USA), 0.5 mg/mL sheared salmon sperm DNA (ThermoFisher Scientific), and 0.5% dextran sulfate (Sigma-Aldrich) in 1× PBS, pH 7.4. Primary antibodies and Ab-oligos were diluted to 15 µg/mL in the same buffer and incubated overnight at 4 °C. The staining solution was removed and the samples were washed with a 2× saline-sodium citrate (SSC) buffer and pH 7.0 (VWR, Radnor, PA, USA) for 3 × 5 min. The samples were incubated with 2% PFA for 15 min to help maintain the retention of antibodies during repeated rounds of cyCIF and to maintain consistent staining methods [29,31]. The samples were then washed with 2× SSC (3 × 5 min). An unconjugated secondary antibody, oligonucleotide-conjugated secondary, or 2× PCL-FL IS were then diluted to 350 nM in an IS dilution buffer containing 2% BSA, 0.5 mg/mL sheared salmon sperm DNA, and 0.5% dextran sulfate in 2× SSC and incubated on the tissues for 45 min, protected from light for the remainder of the staining procedure. The excess IS was removed and the slides were washed with 2× SSC (3 × 5 min). For samples incubated with an oligonucleotide-conjugated secondary, an additional 45 min incubation with the complementary 2× PCL-FL IS at a 350 nM final concentration was performed to complete immunostaining, followed by washing (3 × 5 min in 2× SSC) to remove excess IS. The tissues were stained with 300 nM DAPI for 10 min, washed with 2× SSC (2 × 5 min), mounted in Fluoromount-G (Southern Biotech, Birmingham, AL, USA), and cover-slipped prior to imaging.

### 4.3. Ab-Oligo Fluorescence Signal Amplification

To validate the method of amplifying fluorescence signal using the Ab-oligos, three groups were used for comparison, including (1) conventional indirect IF, (2) Ab-oligo + 2× PCL-FL IS, and (3) an Ab-oligo + 198 nt amplification strand (AmpS), which was detected using 15 nt fluorophore-labeled amplification IS (Amp IS). Each Amp IS was complementary to the AmpS and labeled with a fluorophore at the 3′ end.

Using A431 fresh frozen mouse xenograft tissues, the previously described Ab-oligo staining procedure was used for each group, where only the final fluorescence labeling steps differed for group (3), explained as follows. The partially complementary AmpS was added at 350 nM and incubated at RT for 45 min. The AmpS was removed and samples were washed in a 2× SSC buffer (3 × 5 min). Then, the Amp IS complementary to the AmpS was added at 350 nM and incubated for 45 min protected from light. The remaining washing, DAPI staining, and cover-slipping steps, as outlined above, were then completed [42].

### 4.4. Amplification IS (Amp IS) Molar Excess Titration

The previously detailed FFPE tissue antibody staining procedure was used to stain the MCF7-HER2 xenograft tissue with a HER2 primary antibody or a HER2 Ab-oligo conjugate. A secondary antibody labeled with AF546 or complementary AmpS diluted in IS dilution buffer to 350 nM was added to tissues stained with a HER2 primary antibody or Ab-oligo conjugate, respectively. Following incubation and washing steps, slides stained with HER2 Ab-oligo were then incubated with AF546-labeled Amp IS diluted in an IS dilution buffer to 350 nM–56 µM. A negative control tissue was also generated for each tested concentration where they were processed through the complete staining protocol, but only a secondary antibody or AmpS + Amp IS was added to the tissues to assess background fluorescence without the addition of the primary antibody [42]. The relative SBR for each tissue was calculated, as previously described, and detailed in the image analysis section [29].

### 4.5. Amplification Strand (AmpS) Heating

MCF7-HER2 xenograft tissue was stained as described above. A conventional indirect sample was used as a positive control using a HER2 primary antibody + 350 nM of an AF546 conjugated secondary antibody. Two test samples were stained with HER2 Ab-oligo and incubated with 350 nM of AmpS. The AmpS was kept at RT for one of the samples or heated at 85 °C for 3 min prior to incubation with the other sample. Heating was performed to decrease the possibility of secondary structure formation, a potential outcome with long, single-stranded oligonucleotides, such as the 198 nt AmpS strands. Both Ab-oligo test samples were subsequently labeled with 350 nM of Amp IS. Fluorescence from negative control samples, including tissues stained with AF546-labeled secondary antibody or AmpS + Amp IS only, were used for comparison [42]. The relative SBR for each tissue was calculated, as previously described, and detailed in the image analysis section [29].

### 4.6. Cell Durability after cyCIF

Human CRC PBMC slides were stained using two methods of cyCIF signal removal, one employing photochemical quenching [30] and the other with our Ab-oligo method and UV treatment. Slides were stained over three rounds to compare cell loss and nuclei integrity following repeated staining and imaging cycles. Specifically, for the photochemical quenching, signal removal slides were stained, as previously described [30,40], and after imaging, slides were soaked in 1× PBS and pH 7.4 for 10–30 min in a glass Coplin jar until the coverslip slid off without agitation. Quenching solution containing 20 mM sodium hydroxide (NaOH) and 3% hydrogen peroxide (H_2_O_2_) in 1× PBS, pH 7.4 was freshly prepared from stock solutions of 5 M NaOH (ThermoFisher Scientific) and 30% H_2_O_2_ (ThermoFisher Scientific), and each slide was placed in 10 mL quenching solution. Slides were quenched under incandescent light for 20 min and then washed (3 × 2 min) in 1× PBS and pH 7.4. Subsequent imaging using a Zeiss AxioScan.Z1 (Carl Zeiss AG, Oberkochen, Germany) in the DAPI channel was performed, followed by coverslip removal and quenching. This process was repeated for a total of three rounds. Nuclei were counted using ZEN Blue analysis software (Zeiss) in four randomly selected regions from each round of staining and the average number of nuclei per round normalized to 50,000 nuclei was compared between the two staining methods.

### 4.7. Flexible Highly Multiplexed Image Generation with Indirect IF and Ab-Oligo cyCIF

HCC827 frozen xenograft tissue sections were stained using the previously described Ab-oligo staining protocol. Each tissue section was covered with an unconjugated pEGFR (Table 1) antibody diluted to a concentration of 15 μg/mL and incubated for 1 h at room temperature. The excess pEGFR antibody was removed and then the tissue was washed with 1× PBS (3 × 5 min). The antibody was then fixed on each tissue section with 2% PFA for 15 min and then washed with 2× SSC (3 × 5 min). pEGFR was labeled with donkey anti-rabbit secondary antibody conjugated to Cy7 (Lumiprobe, Hunt Valley, MD, USA) diluted in an IS dilution buffer to 350 nM. Each tissue section, including the negative control, was incubated with diluted secondary antibody for 45 min in the dark. The excess secondary antibody was removed and then the tissue was washed with a 2× SSC buffer (3 × 5 min). Then, to facilitate flexible Ab-oligo cyCIF imaging, the nine Ab-oligos were mixed at a concentration of 15 μg/mL per antibody into a single master mix. Each tissue section was incubated with the diluted antibody cocktail at 4 °C overnight. The next day, the sections were washed with 2× SSC (3 × 5 min). The Ab-oligos were fixed again in 2% PFA for 15 min, then washed again in 2× SSC (3 × 5 min). To facilitate cyCIF imaging, the Ab-oligos were labeled using two different strategies. The first was performed as previously described, where complementary IS labeled with AF488, AF546, or AF647 were utilized [29]. The second method employed the amplification strategy described herein, where AmpS + Amp IS were incubated on tissues at 350 nM and 7 µM, respectively, for each marker. Of note, data are displayed herein demonstrating that 7 µM (20× molar excess compared to AmpS) was the optimal Amp IS concentration.

Cycles of imaging and signal removal were performed using both Ab-oligo fluorescence strategies simultaneously. The strategy using 2× PCL-FL IS was applied for labeling E-Cad, Ki67, CK8, EGFR, and PI3K. The Ab-oligo amplification strategy was used to label pAkt, CC3, pMEK, and Akt. First, AmpS for all markers to be imaged in a given round were diluted to 350 nM in an IS dilution buffer. The AmpS mixture was then heated to 85 °C for 3 min. Subsequently, the 2× PCL-FL IS used in that round were added to the mixture to a final concentration of 350 nM of each 2× PCL-FL IS. The AmpS and 2× PCL-FL IS were incubated and washed, as described above. Amp IS was then diluted to a final concentration of 7 μM and dispensed onto each tissue, followed by the aforementioned incubation and washing steps. The samples were counterstained with DAPI prior to washing, mounting, and cover-slipping.

Images were collected on a Zeiss AxioScan.Z1 in the DAPI, AF488, AF546, AF647, and AF750 channels. All stained slides were then treated with UV light for 15 min followed by washing 10 times with 0.1× SSC, mounting, and cover-slipping. Finally, the slides were imaged with the same settings and in the same FOV used prior to UV treatment to confirm complete signal removal. Subsequent rounds of IS addition, imaging, and signal removal were repeated until all Ab-oligos were imaged. After UV light treatment of the final Ab-oligo imaging round, directly labeled MEK-AF488 and CD45-AF647 were stained onto the tissues and imaged. These antibody targets were selected to be imaged with fluorophore-labeled antibodies because they could not be readily labeled with oligonucleotides.

### 4.8. Fluorescence Microscopy of cyCIF Stained Samples

Cells stained in 96 well plates were imaged on a Zeiss AxioObserver microscope with an AxioCam 506 camera (Carl Zeiss AG, Oberkochen, Germany). Filter sets were selected based on the IS-labeled fluorophore excitation and emission characteristics. Three channels were used for fluorescence image capture, specific to fluorophore-labeled IS, and include the following Zeiss filter sets: EGFP (FITC/Cy2), Cy3/TRITC, and Cy5. The following bandpass (BP) filters were used to filter excitation light: 470/40 (PF), 545/25 (PF), and 620/60 (PF) for the fluorophores Alexa Fluor 488 (AF488), AF546, and AF647, respectively, which were used to label the IS. The following BP filters were used to filter emission light: 525/50, 605/70, and 700/75 for AF488, AF546, and AF647, respectively. To collect DAPI images, excitation light was filtered using a Zeiss 405/40 BP filter. All images collected on the AxioObserver were at 40× (Plan-Apochromat, 0.95A) magnification. Frozen and FFPE tissue sections were imaged on a Zeiss AxioImager.M2 microscope equipped with an XY motorized scanning stage (Zeiss) and a 16-bit Hamamatsu FLASH 4.0 LT+ camera with a Colibri 7 light source (Zeiss). For frozen and FFPE tissue experiments imaged on the AxioImager.M2 microscope, the excitation and emission light were filtered, as previously described, and images were collected at 20× (Plan-Apochromat, 0.8A) magnification [29]. HCC827 xenograft highly multiplex cyCIF tissues and PBMC slides were scanned on the Zeiss AxioScan.Z1 with a Colibri 7 light source (Zeiss) equipped with the following filter cubes: DAPI (Semrock, LED-DAPI-A-000), AF488 (Zeiss 38 HE), AF555 (Zeiss 43 HE), AF647 (Zeiss 50), and AF750 (Chroma 49007 ET Cy7). Exposure times for each channel were determined individually per antibody to optimize dynamic range without image saturation and were kept consistent between conditions. Full tissue scans were taken with the 20× objective (Plan-Apochromat 0.8NA WD = 0.55, Zeiss) and stitching was performed in Zen Blue image acquisition software (Zeiss).

### 4.9. Fluorescent Image Collection, Visualization, and Analysis

All images were collected using the full dynamic range of the microscope camera. All images were acquired with an exposure time sufficient for signal detection, but pixel saturation was prevented. For staining-specific visualization, images were displayed using auto-contrast, where brightness and contrast settings were adjusted to provide optimal visualization of the positive signal. For qualitative image comparison, normalized images were all set to equivalent brightness and contrast settings across an experiment, permitting visual assessment of signal intensities of different staining conditions. Quantification was performed on raw images collected using the full dynamic range of the camera, whereas in a given experiment, images were collected using equivalent image acquisition parameters (i.e., camera settings, magnification, exposure time, etc.). All images displaying DAPI are composites of the relevant fluorescent channel overlaid with DAPI. Where negative controls (e.g., secondary-only or IS-only) have been included in the paper, the relevant fluorescent channel, as well as DAPI, are both shown, although the relevant fluorescent channel may not be bright enough to be visualized when displayed with equivalent contrast settings as other images in the experiment.

For the signal-to-background ratio (SBR) quantification, the fluorescence signal intensity was defined as the mean fluorescent signal per image for the relevant fluorescent stained channels, as measured using ImageJ v1.51. SBR calculations were performed to quantify differences between staining and signal removal conditions, as previously described [29]. For the optimized DS design study, the mean fluorescence intensity, measured using ImageJ, of each image was first normalized to exposure time, and then the relative fluorescence intensity (RFI) was calculated by dividing the normalized fluorescence intensity of all images by that of the brightest image in the experiment. In the study evaluating the oligo-conjugated secondary, QiTissue software [29,31] was used to segment and threshold positive stained samples before and after UV treatment. Negative control samples, secondary-only and IS-only, were segmented but no threshold to identify positive pixels was applied, as there was no detectable fluorescence signal in these images. The RFI was calculated for these images as described above.

## 5. Results

### 5.1. Amplification IS (Amp IS) Titration for the Optimal SBR

**Figure 2:**
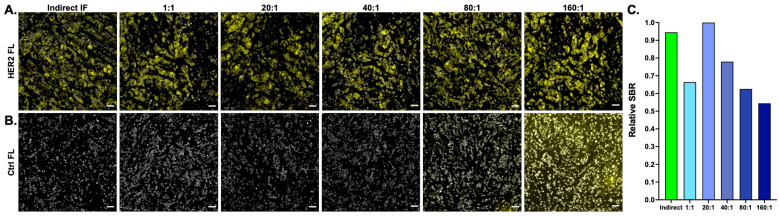
**Ab-oligo amplification molar ratio titration.** (**A**) MCF7-HER2 xenograft FFPE tissue sections were stained with either a HER2 primary antibody for indirect IF or a HER2 Ab-oligo conjugate (HER2 = yellow and DAPI = white), where a titration of molar excess of Amp IS to AmpS was performed from 1:1–160:1. (**B**) Negative controls for each sample were stained with only fluorophore-labeled secondary antibodies or the respective concentrations of AmpS and Amp IS without an Ab-oligo conjugate. (**C**) Image quantification was performed to calculate the relative signal-to-background ratio (SBR) for all staining conditions. Images shown are representative of multiple fields of view and quantification results are reported as a representative average. Scale bar = 50 μm.

Optimal Ab-oligo amplification oligonucleotide staining concentrations were evaluated on HER2 positive (HER2+) tissues (Figure 2) where a titration of the molar ratio of AmpS to Amp IS was performed from 1:1–1:160 and compared to conventional indirect IF HER2 staining. All stained samples resulted in HER2-specific staining patterns (Figure 2A). However, in the negative control samples, which contained neither a primary antibody nor an Ab-oligo conjugate, background fluorescence increased with increasing concentrations of AmpS + Amp IS, demonstrating that increasing concentrations of Amp IS contributed to increased background fluorescence and will decrease the SBR. Notably, the background fluorescence was substantially increased in samples stained with 80× and 160× molar excess of Amp IS to AmpS (Figure 2B). Quantification of the HER2 SBR of the varied staining conditions showed the highest SBR was generated in the tissue stained where the Ab-oligo conjugate was labeled with AmpS and a 20× molar excess of Amp IS (Figure 2C). The next highest SBR was generated using conventional indirect IF-stained tissue, which produced a relative SBR ~6% lower than the 20× molar excess Amp IS staining strategy. As expected from a qualitative evaluation of the images, the SBR decreased in tissues stained with molar excess >20× of Amp IS.

### 5.2. Amplification Strand (AmpS) Heating to Decrease Secondary Structures

**Figure 3:**
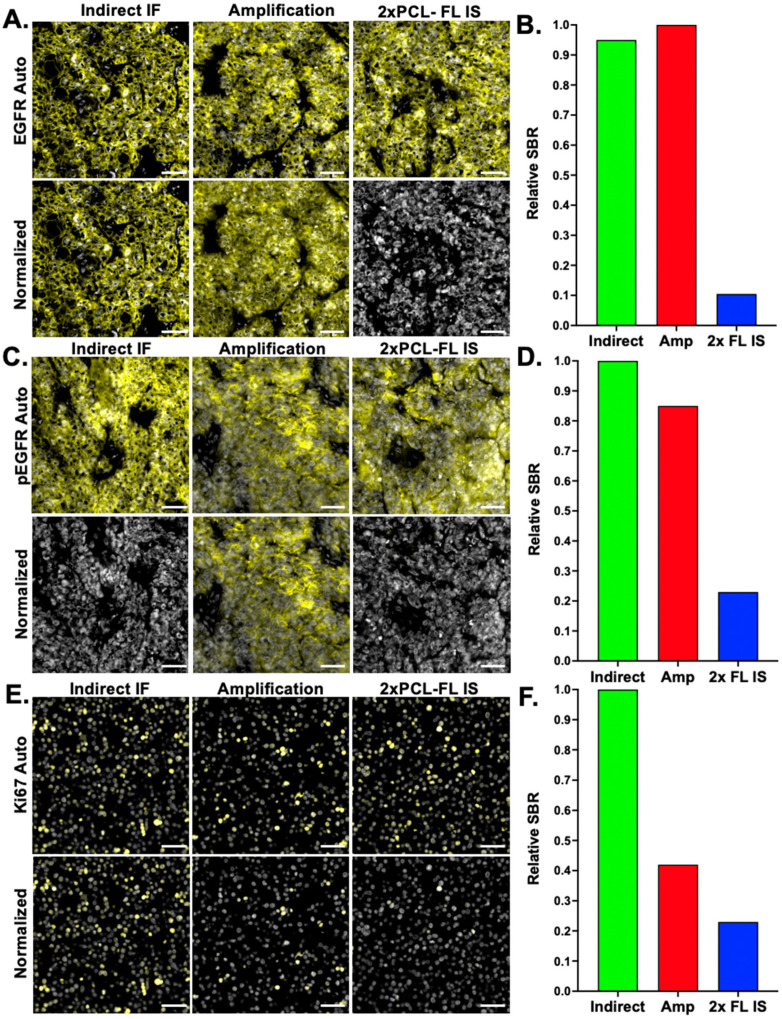
**Ab-oligo amplification strategy evaluation**. The staining pattern and the signal-to-background ratio (SBR) produced by the Ab-oligo amplification strategy was assessed by comparing it to conventional indirect IF and the Ab-oligo conjugate detected using the complementary imaging strand with the 2× PCL-FL IS in A431 mouse xenograft tissues. (**A**) An EGFR-specific staining pattern (EGFR = yellow and DAPI = white) was verified for all staining methods and (**B**) signal intensity was quantitatively assessed for this membrane-bound marker. Similarly, (**C**) the pEGFR staining pattern (pEGFR = yellow and DAPI = white) and (**D**) signal intensity quantification was performed to assess the amplification of a cytosolic protein. (**E**) The Ki67 staining pattern (Ki67 = yellow and DAPI = white) and (**F**) signal intensity quantification were also evaluated for the amplification of a nuclear marker. Staining pattern verification images are displayed with auto-contrast settings for optimal visualization (Auto) and with normalized signal intensity per antigen to compare staining intensity between methods (Normalized). Images shown are representative of multiple fields of view and quantification results are reported as a representative average. Scale bar = 50 μm.

**Figure 4:**
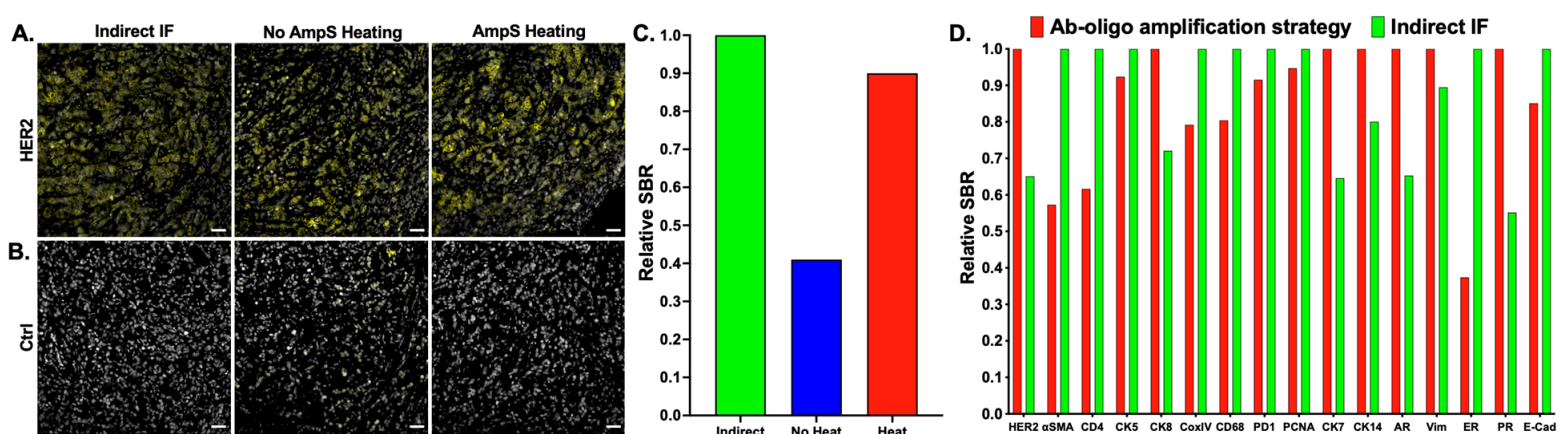
**Amplification strand (AmpS) heating.** (**A**) MCF7-HER2 xenograft FFPE tissue sections were stained with either a HER2 primary antibody for indirect IF or a HER2 Ab-oligo conjugate (HER2 = yellow and DAPI = white), where AmpS was either kept at room temperature prior to application or heated to 85 °C prior to staining. (**B**) Negative controls for each sample were stained with only a fluorophore-labeled secondary antibody or the respective AmpS staining conditions and equivalent Amp IS concentrations. (**C**) Image quantification was performed to calculate the HER2 signal-to-background ratio (SBR) for indirect IF and the Ab-oligo amplification strategy with and without heating of the AmpS prior to staining. (**D**) Indirect IF was compared to the Ab-oligo amplification strategy (20× Amp S to AmpS) using 85 °C heating across antigens including HER2, alpha-smooth muscle actin (α-SMA), CD4, cytokeratin 8 (CK8), CoxIV, CD68, PD1, PCNA, CK7, CK14, androgen receptor (AR), Vimentin (VIM), estrogen receptor (ER), progesterone receptor (PR), and E-Cadherin (E-Cad). Images shown are representative of multiple fields of view and quantification results are reported as a representative average of all images. Scale bar = 50 μm.

While staining HER2+ tissues showed a similar SBR between conventional indirect IF and the optimized AmpS stained with 20× molar excess Amp IS, staining intensity variation was seen when additional antigens were evaluated using this staining methodology (Figure 3). Notably, comparison between labeling Ab-oligo conjugates using either complementary IS with 2 fluorophores (i.e., 2× PCL-FL IS) or the amplification strategy always showed an improved SBR with the amplification strategy for antigen targets regardless of subcellular location (i.e., membrane—EGFR, cytosol—pEGFR, and nucleus—Ki67). However, the relative SBR of the amplification Ab-oligo strategy vs. indirect IF showed substantial variation (Figure 3). Due to the length of the AmpS strands (198 nt), it was hypothesized that secondary structures could form along the AmpS strand, decreasing either the ability of the AmpS to hybridize to the DS or the ability of the Amp IS to hybridize to the AmpS oligonucleotide sequences. To test this hypothesis, MCF7-HER2 xenograft FFPE tissues were stained with a HER2 Ab-oligo conjugate and labeled with AmpS that was either kept at room temperature prior to tissue incubation or AmpS that was heated to 85 °C for 3 min prior to tissue incubation (Figure 4). To facilitate a direct assessment of improved hybridization, a 1× molar excess of Amp IS was used to label each sample after AmpS incubation. The Ab-oligo conjugate-stained tissues were compared to tissues stained with conventional HER2 indirect IF as a positive control. All stained samples generated a HER2-specific staining pattern (Figure 4A), although some visible background was present in the Ab-oligo negative control where the AmpS was not heated prior to tissue incubation (Figure 4B). Relative SBR quantification showed that indirect IF had the highest SBR, while the Ab-oligo-stained tissue with the heated AmpS showed a ~10% lower SBR. Notably, the Ab-oligo-stained tissue with the RT AmpS showed a ~60% lower SBR (Figure 4C). This trend was assessed across antigens and substantial variation was still observed between conventional indirect IF and amplification of the Ab-oligo strategy using heated AmpS as quantified using a relative SBR (Figure 4D).

### 5.3. The Optimization of a DS Oligonucleotide Sequence Design to Decrease Amplified Ab-Oligo Staining Variation

**Figure 5:**
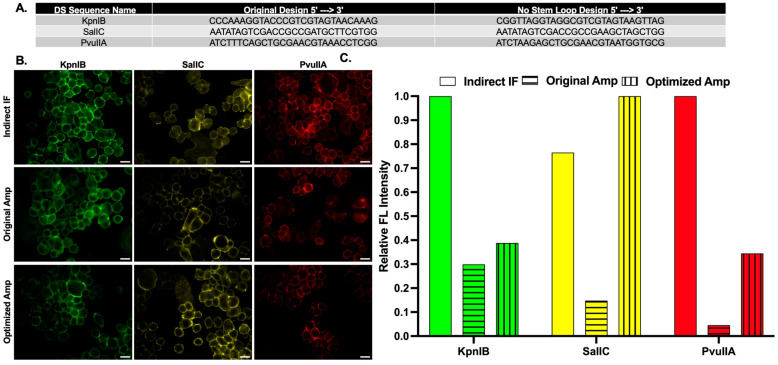
**Optimized docking strand (DS) sequence design.** (**A**) Three DS were evaluated for secondary structure formation and redesigned to reduce these potential structures. (**B**) Fixed cells were stained for HER2 using conventional indirect IF and HER2 Ab-oligo conjugates containing either the original or optimized KpnIB (green), SalIC (yellow), and PvuIIA (red) DS sequence. All images are set to auto-contrast settings for visualization of HER2-specific staining patterns. (**C**) Relative fluorescence intensity was quantified for each image for antibody staining comparison between indirect IF, the original DS sequence design, and the optimized DS sequence design. Images shown are representative of multiple fields of view and quantification results are reported as a representative average of all images. Scale bar = 50 μm.

Given the variation seen across antigen targets (Figure 4D), the specific sequence design of the labeling AmpS was evaluated to determine if certain sequences were more likely to form secondary structures than others. To control for any differences in antibody labeling or location that could affect secondary structure formation, the Ab-oligo amplification strategy was assessed using trastuzumab Ab-oligo conjugates (i.e., HER2 targeted staining), where new AmpS designs for the AmpS sequences led to the generation of complementary, optimized DS. The new DS, termed KpnIB, SalIC and PvuIIA, was compared to the original sequences (Figure 5A). HER2+ SkBr-3 cells were stained using the trastuzumab antibody via indirect IF, an Ab-oligo conjugate with the original DS, and an Ab-oligo conjugate with the new DS. All trastuzumab-stained samples shown HER2-specific staining (Figure 5B). Quantification of the relative fluorescence intensity for each image revealed that indirect IF fluorescence intensity was higher than all but one of the samples, the optimized SalIC DS. However, as expected, all optimized DS designs generated improved relative fluorescence intensity using the Ab-oligo amplification strategy over the original DS designs using the Ab-oligo amplification strategy (Figure 5C).

### 5.4. Oligonucleotide-Labeled Secondary Antibodies Permit Ab-Oligo cyCIF of Sensitive Antibodies

**Figure 6:**
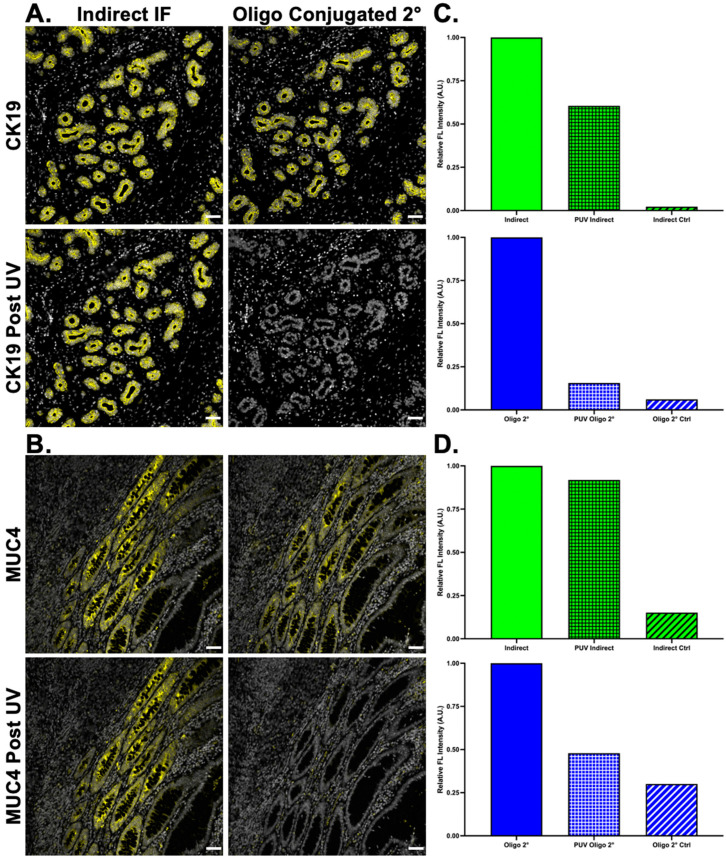
**Oligonucleotide conjugation to secondary antibodies.** Secondary antibodies were conjugated to unique DS and used to stain (**A**) CK19 (yellow) and (**B**) MUC-4 (yellow) in tissues with DAPI (white) staining to show tissue patterns. The oligonucleotide-conjugated secondary antibody staining pattern was compared to indirect IF. Following imaging, both samples were treated with UV light, where the oligonucleotide-conjugated secondary antibodies showed substantially decreased staining. The staining intensity (green) and signal removal (blue) were quantified for both (**C**) CK19 and (**D**) MUC4. Images shown are representative of multiple fields of view and quantification results are reported as a representative average of all images. Scale bar = 50 μm.

While a variety of primary antibodies have been successfully conjugated to designed DS sequences, a subset of antibodies was found to be sensitive to oligonucleotide modification. To alleviate this challenge, DS was conjugated to secondary antibodies, permitting the labeling of these sensitive primary antibodies in the first round of Ab-oligo cyCIF studies (Figure 1D). As a proof-of-concept, CK19 (a robust antibody that tolerates oligonucleotide conjugation well) and MUC4 (an antibody that precipitated upon oligonucleotide conjugation) primary antibodies were stained using conventional indirect IF and oligonucleotide-conjugated secondary antibodies on serial tissue sections. Both antibodies demonstrated similar staining patterns using either conventional indirect IF or Ab-oligo indirect IF staining methods (Figure 6A,B). Notably, UV light treatment of the samples stained with the oligonucleotide-conjugated secondary antibodies facilitated the removal of the marker-specific staining pattern using the same 2× PCL-FL IS strategy as with the Ab-oligo labeled primary antibody conjugates. UV light treatment decreased the relative fluorescence intensity in the conventional indirect IF-stained tissues, likely due to fluorophore photobleaching, but the marker-specific staining pattern persisted. However, as expected, the fluorescence signal intensity of oligonucleotide-conjugated secondary antibodies was reduced to near background levels after UV treatment, as measured by the control sample and the absence of a staining pattern (Figure 6C,D).

### 5.5. Ab-Oligo cyCIF Decreases Cell Loss Compared to Photochemical Quenching cyCIF Techniques

**Figure 7:**
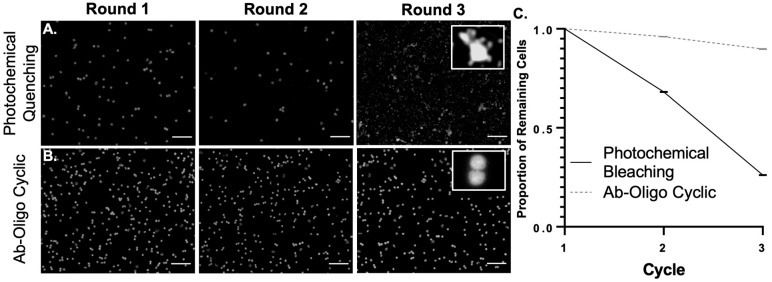
**Single Cell Sample Integrity after cyCIF.** PBMCs were stained with DAPI (white) and subject to the staining, imaging, and signal removal conditions for (**A**) photochemical quenching and (**B**) Ab-oligo cyCIF. The nuclear staining is shown in a representative region of interest after rounds 1–3, where the inlaid image shows the nuclear morphology after round 3. (**C**) DAPI nuclear staining was used to quantify the proportion of remaining cells for photochemical bleaching and Ab-oligo cyCIF after rounds 1–3. Images shown are representative of multiple fields of view and quantification results are reported as a representative average of all images. Scale bar = 50 μm.

Tissue and cell loss as cyCIF round number increases has been a challenge for a variety of cyclic immunostaining techniques, particularly the fluorophore photochemical quenching and antibody stripping techniques that require harsh tissue treatments [43,44]. This is especially problematic in samples with a minimal extracellular matrix and pronounced in single-cell samples such as blood smears. The ability to preserve cells in blood smear samples following conventional bleaching cyCIF vs. our Ab-oligo cyCIF was directly compared over three rounds of staining using DAPI to quantify cell durability. Photochemical quenching cyCIF showed substantial cell loss and change in nuclear morphology after only three rounds of cyCIF, rendering the sample unusable for subsequent analyses (Figure 7A). By comparison, minimal cell loss or nuclear shape change were seen over three rounds of staining using the Ab-oligo cyCIF method (Figure 7B). The proportion of remaining cells was quantified for both photochemical quenching (round 2 = 34,449 and round 3 = 6667) and Ab-oligo cyCIF (round 2 = 48,017 and round 3 = 44,906), and was found to be substantially higher using Ab-oligo cyCIF (Figure 7C).

### 5.6. Highly Multiplexed Image Generation with Ab-Oligo cyCIF and Indirect IF

**Figure 8:**
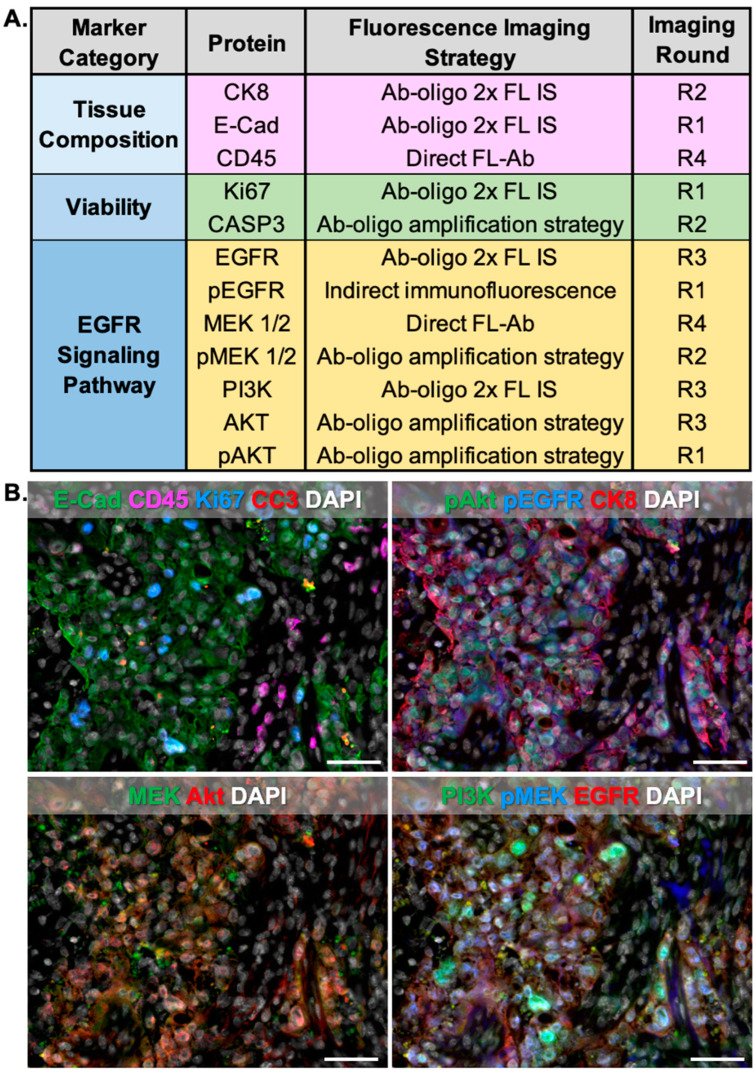
**Highly multiplexed flexible cyCIF.** (**A**) Indirect IF was combined with Ab-oligo cyCIF using 2× PCL-FL and amplification strategies followed by a final round of direct IF staining to generate a 12-color image on the frozen HCC827 xenografts. (**B**) A representative region of interest is shown to demonstrate that these strategies can be readily combined to generate spatial proteomic data. Scale bar = 50 μm.

Unconjugated primary, directly conjugated primary, and Ab-oligo conjugates (Table 1) were used to generate a 12-color image on a frozen HCC827 xenograft tissue using a flexible approach to cyCIF by combining different staining strategies (Figure 8). Unconjugated primary antibodies for pEGFR and AF750 labeled secondary, respectively, were applied alone as a first round of staining. The master-mix of Ab-oligo conjugates was then incubated in a single staining step followed by IS applied in groups of three using either 2× PCL-FL IS or the Ab-oligo amplification strategy. Specifically, E-Cad, Ki67, CK8, EGFR, and PI3K were imaged using the 2× PCL-FL IS strategy while pAkt, CC3, pMEK, and Akt were imaged using the optimized Ab-oligo amplification strategy. Finally, MEK and mouse-specific CD45 were imaged with fluorophore-labeled antibodies (Figure 8A). A mouse-specific CD45 antibody was selected to only label mouse immune cells. No colocalization with CK8, a marker for human epithelial cells, was observed, which provided evidence of the mouse antigen specificity of CD45. Tissue scanning was performed in each staining round for visualization of the specimen with one representative field of view displayed where individual cell staining patterns are shown (Figure 8B). A marker-specific staining pattern was seen for all antibodies imaged regardless of staining method, where indirect IF staining for pEGFR was successfully performed before any Ab-oligo conjugate was present on the tissue. Additionally, both the 2× PCL-FL IS and the Ab-oligo amplification strategy were employed simultaneously in a single round of imaging without any deleterious effects on accurate antigen labeling. Due to the relative homogeneity of xenograft tissues, there was overlap in the staining pattern for some of the markers, particularly for the EGFR signaling pathway proteins EGFR, pEGFR, Akt, pAkt, PI3K, and pMEK. E-Cad and CK8, commonly expressed in epithelial cells, provided a spatial map of the HCC827 NSCLC cells. Further, Ki67 and CC3, marking proliferating and apoptotic cells, respectively, were expressed in distinct cells from one another where DAPI staining provided spatial context (Figure 8B).

## 6. Discussion

A variety of antibody staining methods and cyclic immunostaining methods have been proposed to facilitate highly multiplexed spatial proteomics [6,7,8,9,10,11,12,13,14,15,16,17,18,19]. However, it is often challenging to apply a single antibody labeling strategy across all desired biomarker targets, as some antibodies are sensitive to changes from conjugation and may lose specificity. Herein, we demonstrate the utility of a more flexible cyCIF strategy by combining our Ab-oligo cyCIF with conventional direct and indirect staining, as well as extend our Ab-oligo cyCIF strategy to label secondary antibodies (Figure 1). Additionally, a critical component of a robust, highly multiplexed imaging platform is detection sensitivity sufficient for the assessment of cell signaling pathway proteins, such as phosphorylated proteins. This is a crucial component of building a comprehensive spatial proteomic characterization of a tumor and its microenvironment. However, both conventional antibody-based and mass spectrometry imaging (MSI) techniques for highly multiplexed image generation lack amplification strategies which limits their sensitivity to perturbations in the signaling pathway, potential detection sensitivity for rare cell subpopulations, and critical but difficult-to-detect changes in cell signaling.

To overcome these challenges, an amplification strategy compatible with the existing Ab-oligo cyCIF methodology was developed and optimized. Optimization of the Ab-oligo cyCIF amplification strategy SBR was first assessed through a titration study to identify the molar excess of Amp IS required to maximize staining intensity while minimizing background (Figure 2). The optimal molar ratio of Amp IS to AmpS was found to be 20:1, which resulted in marker specific staining pattern with no qualitatively visible background fluorescence (Figure 2A). Additionally, no background was visible in the control tissue for the tested 20:1 molar ratio when the negative control image was set to the same contrast settings as the corresponding stained tissue (Figure 2B). Notably, further increase in Amp IS to AmpS caused increased background in the control samples that were not antibody stained, with a visible background in the 80:1 and 160:1 Amp IS to AmpS. This increased background fluorescence was likely due to nonspecific Amp IS tissue sticking that could be attributed to either the IS or the fluorophore, or both. Furthermore, a 20× molar excess of Amp IS produced an SBR slightly greater than that of indirect IF (Figure 2C). While a rather high molar excess was selected, representing increased reagent cost, the 1× molar excess also produced marker-specific staining. Further titration on a per-marker basis is ongoing to determine if lower optimal concentrations could be used for some antigens to reduce reagent costs.

While the amplification strategy improved the SBR for the test biomarker HER2, this improvement in the SBR was found to be variable across biomarkers (Figure 3). It was hypothesized that variation may stem from the secondary structure formation of the amplification strand given its relatively long length (198 nt), decreasing efficient hybridization with either the DS conjugated to the antibody or the Amp IS used for visualization. Two strategies were investigated to minimize secondary structure formation. First, AmpS heating was employed to remove any secondary structures prior to sample staining (Figure 4). It was observed that heating AmpS to 85 °C for 3 min prior to tissue incubation improved the SBR (Figure 4A–C), a step common in protocols for polymerase chain reaction (PCR) assays for the denaturation of oligonucleotide templates prior to measurements [45]. While heating was integrated into the amplification the Ab-oligo cyCIF protocol, variation in staining intensity compared to conventional indirect IF was still observed across a panel of stained biomarkers (Figure 4D). Additionally, this evaluation of amplification performance across markers also indicated that not all markers require amplification, where more abundant antigens, such as cytokeratin markers (e.g., CK5, CK8, and CK19), could be readily imaged using the 2× PCL-FL IS, decreasing overall reagent cost.

This result prompted further investigation into the design of the AmpS oligonucleotide sequences in an effort to reduce the probability of secondary structure formation. Through evaluation of oligonucleotide sequence composition, it was determined that the region of the AmpS complementary to the Ab-oligo labeled DS had a high potential to form secondary structures that can form when two or more regions of the same strand are complementary to one another [46]. Three AmpS sequences, KpnIB, SalIC, and PvuIIA, were all found to possess a high probability for stem-loop secondary structure formation. These AmpS sequences were re-designed to decrease the probability of secondary structure formation in the portion of the AmpS sequence that is complementary to each of these DS sequences (Figure 5A). After conjugating to a HER2-specific antibody to compare staining patterns with the original DS, both the original and re-designed DS sequences showed marker-specific staining patterns when stained with the Ab-oligo amplification strategy (Figure 5B). However, Ab-oligo conjugates with all three new DS designs showed higher relative fluorescence intensity than their respective original designs (Figure 5C). This resulted in increased attention to future AmpS sequence design for subsequent Ab-oligo conjugates to mitigate secondary structure formation potential.

To increase the flexibility of the Ab-oligo cyCIF strategy, secondary antibody oligonucleotide labeling was investigated. Given the sensitivity of some primary antibodies to conjugation, the addition of a 28 nt oligonucleotide sequence can substantially change their staining specificity, making their integration into a cyCIF panel difficult. While indirect IF can be used in the first round of a cyCIF study without compromising the ability to further stain the tissue, its specific signal cannot be readily removed with UV light treatment (Figure 6A,B). However, with the addition of oligonucleotide labeled secondaries, indirect IF can be utilized as a first round of staining, where complementary 2× PCL-FL IS are used to fluorescently label Ab-oligo secondary antibodies which have bound to the unconjugated primary antibody. Notably, the signal can be readily removed using UV light treatment rendering the tissue fluorescence levels similar to autofluorescence for subsequent cyCIF staining (Figure 6C,D).

While tissue samples can be stained using a variety of cyclic immunostaining strategies, fragile samples, particularly those without an extracellular matrix, such as single cells, have been found sensitive to the relatively harsh conditions used for photochemical quenching or antibody stripping. Notably, signal removal using the combination of PCLs and UV light readily demonstrated single-cell preservation in blood smears which are substantially damaged by photochemical quenching after only three rounds of cyCIF (Figure 7). Thus, a cyCIF staining strategy that employs gentle signal removal, such as UV light cleavage, is desirable to extend cyCIF to a variety of sample types.

A key benefit to Ab-oligo cyCIF is its flexibility to alter the imaging strategy on a per-marker basis to accommodate markers where oligonucleotide conjugation was unsuccessful and markers that needed Ab-oligo amplification could be readily incorporated into the Ab-oligo cyCIF imaging methodology. Herein, we demonstrate that markers stained by indirect IF, antibodies directly labeled with a fluorophore (direct IF), the 2× PCL-FL IS Ab-oligo strategy, or the Ab-oligo amplification strategy can be imaged sequentially and/or simultaneously on the same tissue section (Figure 8). Validation of the flexibility of our Ab-oligo cyCIF platform resulted in the generation of 12-color images on HCC827 xenograft tissue sections, where conventional indirect IF along with the Ab-oligo imaging strategies using the 2× PCL-FL IS or the amplification Ab-oligo strategies were imaged simultaneously followed by direct IF as a final round of staining (Figure 8A). pEGFR was labeled via indirect IF, where it was stained first to avoid cross-reactivity of the fluorophore-labeled secondary antibody between antibodies of the same host species. This first antibody staining step did not disrupt the subsequent staining of Ab-oligo conjugates where cycles of three Ab-oligo conjugates were imaged per round. The first round of imaging included three Ab-oligo conjugates along with pEGFR, but since pEGFR was stained with conventional IF, the signal could not be removed with UV light treatment. Thus, all subsequent rounds of imaging were completed by excluding the AF750 channel (i.e., the channel in which pEGFR was imaged) with three markers per round (i.e., AF488, AF546, and AF647). Importantly, no interactions occurred between the 2× PCL-FL IS and the AmpS that were co-incubated on the samples, potentially in part due to the heating of the AmpS that denatured potential complexes formed between the different oligonucleotides. For example, the staining patterns of cleaved caspase 3 (CC3) and CK8, which were stained using the amplification and 2× PCL-FL IS strategies, respectively, were found to maintain specificity (Figure 8B). The final round of imaging was performed with directly labeled antibodies targeting CD45 and MEK. In the representative images, there are CD45-positive cells in a spatially distinct area from cells labeled for E-Cadherin or CK8, which serve as tumor cell markers. The selected CD45 antibody was reactive with mouse tissue only in this model system which will not be positive for tumor markers, as the tumors are a human NSCLC cell line. This provided support to the conclusion that the CD45 was labeling mouse immune cells in the non-tumor/mouse tissue infiltrate regions of the tissue sections. Further, MEK was observed to label tumor cells as expected in this tissue. This serves as validation of the flexibility of the Ab-oligo cyCIF imaging methodology.

One limitation of the presented multiplexed cyCIF study was utilizing a combination of reagents that facilitated signal removal using UV light (Ab-oligo strategies) with those that did not (indirect and direct IF strategies). Due to these limitations, once indirect or direct IF reagents were used for sample staining, no further imaging was performed in those channels. Therefore, to further advance the flexibility of the cyCIF platform, future multiplexed imaging studies will investigate the integration of additional signal removal techniques to maximize assay speed by maintaining all imaging channels for cyCIF rounds. Additionally, in lieu of conventional indirect IF reagents, future studies will also investigate further use of the oligonucleotide-conjugated secondary antibodies, where secondary antibody amplification of signal will be achieved, but then readily removed with the conventional Ab-oligo cyCIF workflow. An additional limitation of this proof-of-concept study was the lack of further delineation of mouse stromal cells that are not labeled by any of the markers in the panel designed for this study. Future studies will include additional mouse-specific antibodies to identify additional mouse cell types (e.g., fibroblasts and endothelial cells) in order to improve the strength of the experimental findings.

## 7. Conclusions

In conclusion, the demonstrated flexibility of the Ab-oligo cyCIF platform opens the possibility for the design and optimization of antibody panels using both low-abundance antigens and antibodies sensitive to conjugation. Routine integration of oligonucleotide-conjugated secondaries, 2× PCL-FL IS Ab-oligo staining, the optimized Ab-oligo optimization strategy, as well as direct and indirect IF, will permit a wide variety of antibody panel staining on tissue and single cell samples without substantial tissue loss to investigate spatial proteomics in health and disease.

## Figures and Tables

**Table 1:** Antibody-specific information.

Protein	Ab Clone	Catalog	Host	Vendor
AKT	40D4	2920	Mouse	CST
pAKT	D9E	4060	Rabbit	CST
𝛼SMA	EPR5368	ab220795	Rabbit	Abcam
AR	ER179(2)	ab212175	Rabbit	Abcam
CC3	D3E9	9579	Rabbit	CST
CD4	EPR6855	ab181724	Rabbit	Abcam
CD45 ^%†^	D3F8Q	70257	Rabbit	CST
CD68	KP1	916104	Mouse	Biolegend
CK5	EP1601Y	ab214586	Rabbit	Abcam
CK7	EPR1619Y	ab18131	Rabbit	Abcam
CK8	EP1628Y	ab217173	Rabbit	Abcam
CK14	LL002	MCA890	Mouse	Bio-Rad
CK19	A53-B/A2	628502	Mouse	Biolegend
CoxIV	3E11	4850	Rabbit	CST
E-Cad	EP700Y	ab201499	Rabbit	Abcam
EGFR	EP38Y	ab174481	Rabbit	Abcam
pEGFR	D7A5	3777	Rabbit	CST
ER	EPR4097	ab167610	Rabbit	Abcam
HER2	3B5	MA5-13675	Mouse	ThermoFisher
Ki-67	polyclonal	ab15580	Rabbit	Abcam
MEK 1+2 ^†^	EPR16667	ab200177	Rabbit	Abcam
pMEK 1	EPR3338	ab214445	Rabbit	Abcam
PCNA	EPR3821	ab218310	Rabbit	Abcam
PD1	EPR4877(2)	ab186928	Rabbit	Abcam
PI3K	EPR18702	ab223792	Rabbit	Abcam
PR	YR85	ab206926	Rabbit	Abcam
VIM	D21H3	5741	Rabbit	CST

^%^ Reactive with only mouse cells, ^†^ Not oligo-conjugated.CST = Cell Signaling Technology, AR = androgen receptor, CC3 = cleaved caspase 3, CK = cytokeratin, E-Cad = E-Cadherin, EGFR = epidermal growth factor receptor, PR = progesterone receptor, VIM = vimentin.

## Data Availability

The data presented in this study are available upon reasonable request from the corresponding author.
